# A Review of the Nonsurgical Treatment of Oral Leukoplakia

**DOI:** 10.1155/2010/186018

**Published:** 2010-02-23

**Authors:** Adriana Spinola Ribeiro, Patrícia Ribeiro Salles, Tarcília Aparecida da Silva, Ricardo Alves Mesquita

**Affiliations:** Department of Oral Surgery, Medicine and Pathology, Dental School, Federal University of Minas Gerais, CEP 31.270-901, Belo Horizonte, Minas Gerais, Brazil

## Abstract

The aim of this paper was to assess the nonsurgical treatment of oral leukoplakia (OL). A medline search from 1983 to 2009 was conducted. The topical or systemic nonsurgical treatments or combination of both was reviewed. The primary outcomes of interest were clinical resolution, malignant transformation, follow-up, and recurrence of OL. Studies showed a rate higher than 50% of clinical resolution with photodynamic therapy, beta-carotene, lycopene, or vitamin A. Few studies reported rates of recurrence from 5 to 67% and of malignant transformation from 8 to 23%. There is a lack of randomized clinical trials that assess the effectiveness of nonsurgical treatment of OL. At this time, randomized controlled trials for nonsurgical treatment of OL demonstrate no evidence of effective treatment in preventing malignant transformation and recurrence. It reinforces that even after clinical resolution, OL should be regularly followed.

## 1. Introduction

Oral leukoplakia (OL) is a premalignant lesion described as “a predominant white lesion of the oral mucosa which cannot be defined as any other known lesion” [1]. According to Warnakulasuriya et al. [2], the new concept of OL shall acknowledge white lesions with questionable risk of being an OL, being excluded any other pathologies or known disorders which do not present potential malignant risk such as candidiasis, lupus erythematosus, lichen planus, hairy leukoplakia, frictional keratosis, nicotinic stomatitis, and leukoedema [3, 4]. 

OL's etiopathogenesis encompasses two broad categories, as follows: OL of unknown etiology or idiophatic and OL associated with tobacco use [3]. OL is more often found among older and elderly men, and its prevalence increases with age advancement. It has been estimated that less than 1% of the affected men are younger than 30 years old and that the prevalence increases to 8% in male patients older than 70 years old and to 2% in female patients of 70 years or more. OL's histopathologic aspects may vary from epithelium atrophy to hyperplasia, which can be associated with varying degrees of epithelial dysplasia [4, 5].

OL located on the floor of the mouth, soft palate, and tongue are considered as high-risk lesions, while, in other areas, they may be considered as of low malignancy risk [5, 6]. OL has an annual malignant transformation rate of 0.1% to 17% [7–10]. Some factors may contribute to increase the chance of the OL becoming malignant [4, 6, 11, 12]; these include the following.

1. Gender: female patients tend to present a higher risk of developing the malignant form [11, 12].
2. A long-time OL lesion: OL resistant to the treatments and what persist for long time may have worse prognosis than recent [12].
3. OL in sites of high risk: lesions in the floor of mouth, ventrolateral tongue and soft palate have a high risk of malignant transformation [6, 12].
4. OL among nonsmokers (idiopathic): nonsmokers with OL have an increased rate of malignant transformation in relation to OL in smokers [11, 12].
5. Nonhomogenous OL-type: nonhomogenous OL lesions have a mixed color of white and red alterations, and an exophytic, papillary, or verrucous aspect; regardless of treatment, they exhibit a high recurrence rate and often eventually transformation to squamous cell carcinoma [4, 12].
6. Epithelial dysplasia: OL with moderate and severe dysplastic lesions had a significantly higher risk of developing a squamous cell carcinoma than OL without epithelial dysplasia or with mild epithelial dysplasia [7, 9, 12].

In order to conduct treatment for OL, the degree of epithelial dysplasia may be assessed. In the presence of moderate or severe epithelial dysplasia, surgical treatment is recommended [9]. However, OL presenting low to moderate malignant risk may be either completely removed or not, and the decision should consider other factors such as location, size and, in the case of smokers, the patient's engagement in smoking cessation [4, 9, 13]. OL surgical treatment may be performed either through conventional surgery [4, 5, 14], electrocauterization, laser ablation [15, 16], or cryosurgery [17, 18]. Recurrence of OL after surgical treatment has been reported in 10%–35% of cases [19–22].

Nonsurgical treatment may also be considered for the management of OL [17, 18, 23]. This modality offers minimal adverse effects to patients, especially for patients with widespread OL that involves a large area of the oral mucosa or patients with medical problems and, consequently, high surgical risks [24]. Additionally, potential advantages of the nonsurgical treatment of OL include easy application that does not require treatment at a medical center and relative low cost [25].

The purpose of this paper is to present the current nonsurgical treatment options for OL. A Medline from 1983 to 2009 search was conducted using the following keywords: nonsurgical treatments of OL, retinoids, carotenoids, lycopene, photodynamic therapy. We included 21 studies of patients with diagnosis of OL. The primary outcomes of interest were clinical resolution, follow-up, and when reported, malignant transformation and recurrence. The topical or systemic nonsurgical treatments or combination of both were reviewed. Furthermore, the mechanisms of action, biodisponibility, toxicity, and side effects of these treatments were analyzed. Table 1 presents the nonsurgical treatment options for OL. 

## 2. Carotenoids

### 2.1. Beta-Carotene

The carotenoids are a group of extremely hydrophobic molecules with little or no solubility in water [26]. Beta-carotene is a carotenoid commonly found in dark green, orange or yellowish vegetables, such as spinach, carrots, sweet potato, mango, papaya, and oranges [27]. Beta-carotene is a vitamin A precursor [26, 28–30]. The only known effect of excessive beta-carotene intake is a state in which the skin becomes strongly yellowish, the so-called carotenodermy, which disappears in a few weeks after the reduction of consumption [26]. While some authors have demonstrated the absence of side effects in patients that have received beta-carotene treatment [27], in other studies, the supplement diet based on beta-carotene caused headaches and muscle pain in some of the patients [31].

The use of beta-carotene has been recommended in order to prevent OL and possibly oral cancer [31]. The potential benefits and protective effects against cancer are possibly related to its antioxidizing action [32–34]. This function is accomplished through a ligation between beta-carotene and oxygen, which is an unstable reactive molecule, thus diminishing the damaging effects of free radicals [34, 35]. According to Liede et al. [32], a diet supplemented with beta-carotene can prevent changes in the oral mucosa, especially in smoker patients, who present low serum levels of vitamin C and beta-carotene when compared to nonsmokers. It has also been shown that beta-carotene has a better therapeutic clinic response in the prevention of OL lesions in smoker patients than in the nonsmoker ones [36]. Results from Sankaranarayanan et al. [31] demonstrated that one third of patients (15 out of 46) that used 360 mg beta-carotene per week during 12 months presented a complete resolution of OL. In the evaluative sessions one year after the treatment, 8 out of 15 (54%) of the patients who had a complete response presented recurrence. Moreover, 12 months after stopping the supplements, 5% (2 out of 15) of the patients who had made use of beta-carotene developed malignant neoplasia adjacent to the OL. Side effects were observed in 5 patients, being 3 with headaches and 2 developed muscular pain.

In another study, 23 patients with OL were treated with beta-carotene, in oral doses of 90 mg/day, for three cycles of 3 months each. Of 18 patients who completed the study, 6 (33.3%) showed complete clinical response. No significant clinical signs of toxicity were detected in any of the patients [37].

Twenty-four patients with OL were employed in another study with beta-carotene employed at a dose of 30 mg/day for six months. Only 2 patients (8.3%) presented a complete clinical response and 15 patients (62.5%) had partial clinical response [38]. Garewal et al. [39] evaluated 50 patients with OL, treated with beta-carotene at a dose of 60 mg/day, for six months. Only 2 patients (4%) demonstrated a complete clinical response. Relapses were found in 4 patients. A second biopsy was obtained after 6 months of therapy in 23 patients. There was no change in the degree of dysplasia in 14, with improvement of at least 1 grade in 9 (39%). 

In the revised studies, the percentage of patients with clinical resolution ranged from 4% to 54%, with dosages regimes from 20 to 90 mg/day of beta-carotene in time periods from 3 to 12 months (Table 1).

### 2.2. Lycopene

Lycopene is a carotenoid without provitamin A action. This is a fat-soluble red pigment found in some fruit and vegetables. The greatest known source of licopene is tomatoes, which are widely employed in cooking [40]. There is a positive relationship between lycopene consumption and a reduction in the risk of the development of degenerative diseases caused by free radicals, such as cancer and cardiovascular diseases [41, 42]. Lycopene has the uncommon feature of becoming bound to chemical species that react to oxygen, thus being the most efficient biological antioxidizing agent [41]. Due this property, studies have been enthusiastically conducted with lycopene, in order to find out whether or not it could be an alternative to protect patients against the damaging effects of free radicals [41]. In addition to its antioxidizing property, lycopene also has the capacity to modify intercellular exchange junctions, and this is considered to be an anticancer mechanism [41]. In vitro experiments have shown the inhibition of the process of human neoplastic cellular growth by lycopene, since this protein interferes in growth factor receptor signaling and, thus, in cellular cycle progression, as previously demonstrated for neoplastic cells from the prostate gland [43].

Consumed carotenoids are incorporated in lipidic micelles and are absorbed by enteric mucosa through passive diffusion and distributed to the organs by the plasmatic lipoproteins. Lycopene release from the food matrix, presence of fat in the diet, and heat-induced isomerization from trans to cis mode are some factors influencing lycopene absortion and biodisponibility [44].

Lycopene is better absorbed in oil resin capsules and in tomato juice than in the form of raw tomatoes [45]. Hoppe et al. [46] determined the biodisponibility of one synthetic type of lycopene, in contrast with the performance of a lycopene oil resin extracted from tomatoes, and concluded that both sources of lycopene have the same bioavailability. Nagao et al. [47] evaluated 48 patients with OL (38 men and 10 women) and 192 control patients to verify the relationship between OL with serum levels of retinol, alpha-tocopherol, zeaxantine and luteine, cryptoxantine, lycopene, and alpha and beta-carotenes. The serum levels of lycopene and beta-carotene, among the 38 men suffering from OL, were significantly lower than those of the control group (*P* < .005). Authors suggested that improvement of micronutrient levels of beta-carotene and lycopene in Japanese males with a high frequency of smoking habit may protect against the relative risk of OL in this population.

Singh et al. [48] assessed the efficiency of lycopene in 58 cases of OL. The patients were divided into three groups, and received 8 mg/day, 4 mg/day, and placebo for a period of three months. The supplementation of lycopene (8 mg/day and 4 mg/day) reduced hyperkeratosis (clinically measured by the size of the lesion) with a similar efficiency in 80% of the cases. The complete clinical response of patients receiving 8 mg/day was 55% and 4 mg/day was 25%, but this difference was not statistically significant.

No systemic significant toxical effect of lycopene has been observed and there is no evidence of side effects from the treatment with lycopene [49]. Lycopene is a promising candidate in reducing cancer and chronic diseases in human beings; however, further research is needed to clarify its potential function in human health, according to the following criteria [50, 51].

1. Factors influencing the uptake of lycopene in the diet, including the way it interacts with other carotenoids.
2. Human metabolism and the possible function of the metabolites and cis-trans isomers.
3. Mechanisms of the direct or indirect modulation of cancer.
4. Studies based on evidences of treatment in human beings.
5. Mechanisms of lycopene deposition in human tissues.
6. Lycopene effects in the immunological system.

Only one study evaluated lycopene in clinical resolution of OL. The time period was three months, the dosages regimes from 4 mg/day and 8 mg/day and patients had clinical resolution 25 and 55%, respectively (Table 1).

## 3. Vitamins

### 3.1. L-Ascorbic Acid (Vitamin C)

L-ascorbic acid (L-AA), the so-called vitamin C, is found in citrous fruits such as kiwi, strawberries, papaya, and mango [52]. The current US recommended daily allowance for ascorbic acid ranges between 100–120 mg/per day for adults [52]. It has been suggested that a daily intake of at least 140 mg/day is required for smokers because they usually present a reduction of the L-AA concentration in serum leukocytes [53].

L-AA has antioxidizing properties and reacts with superoxide produced as a result of the cells' normal metabolic processes; this inactivation of superoxide inhibits the formation of nitrosamines during protein digestion and helps avoid damage to DNA and cellular proteins [54]. L-AA toxicity does not occur, since vitamin is water-soluble and a decrease in absorption efficiency occurs when consumption exceeds 180 mg/day [55].

The ability of L-AA to maintain oral mucosa integrity is very little documented. One study examined the presence of oral mucosal lesions in subjects with low L-AA levels in plasma, compared to controls. Subjects with low plasma L-AA levels ≤25 *μ*mol/l) (*n* = 106) formed the study group and individuals with normal L-AA levels (≥50 *μ*mol/l) (*n* = 103) formed the control group. Oral mucosal lesions in all subjects were defined clinically as petechias, OL, and lichenoid lesions. There was a statistically significant difference between the groups only for OL, where the prevalence of OL was higher when smoking was combined with L-AA deficiency [56].

In another study [57], 24 OL patients were treated with an association of beta-carotene, vitamin E, and L-AA, and an increase was observed in the reversion of oral mucosa dysplasia. In 97.5% of patients, dysplasias were diminshed by use of antioxidant combinations. The reversion of the oral mucosa dysplastic changes was more evident in the patients using antioxidative vitamins that stopped smoking and ingestion of alcohol. 

There are no studies regarding the efficacy of the use of L-AA alone for OL treatment.

### 3.2. *α*-Tocoferol (Vitamin E)

*α*-Tocoferol (AT) is the commonest and most active form of vitamin E. It is found in plant oil, margarine, and green leaves [58–61]. The recommended daily limit rates are 10 mg/day for adult men and 8 mg/day for adult women [62]. Its absorption rate is reduced when consumption exceeds 30 mg/day [63]. *α*-Tocoferol is an effective antioxidant at high levels of oxygen, protecting cellular membranes from lipidic peroxidation [59, 60, 64, 65]. Erhardt et al. [35] showed that supplementation with AT led to a significant rise in the concentration of this antioxidant in the plasma. In contrast, supplementation did not lead to a significant increase in the concentration of AT in cells of the oral mucous membrane.

Benner et al. [66] evaluated the toxicity and efficacy of AT in 43 patients with OL in use of 400 IU twice daily for 24 weeks. Follow-up was performed at 6, 12, and 24 weeks after the beginning of treatment to assess toxicity, clinical response, and serum AT levels. It was observed that 10 patients (23%) had complete clinical remission of lesion and 10 (23%) had a partial clinical response. Nine (21%) had histologic responses (complete reversal of dysplasia to normal epithelium). Mean serum AT levels were 16.1 *μ*g/mL at baseline and increased to 34.29 *μ*g/mL after 24 weeks of treatment. 

Seventy-nine patients with OL were enrolled in an antioxidant supplementation program that consisted of 30 mg of beta-carotene, 1000 mg of L-AA, and 800 IU of AT per day, which were taken for 9 months. No side effects were noticed during the course of the study. Although no patients showed complete resolution of the OL, 55.7% showed reduction in the size of the OL after 9 months. Clinical improvement was observed in 90% of the patients who had reduced risk factors, compared with 48.8% of improvement in those who did not. Squamous cell carcinoma developed in seven patients (8.9%) within the preexisting OL during or shortly after completion of the study [33].

Some studies evaluated the administration of AT, alone or combined, used the dosage of 800 IU/day from 6 to 9 months (Table 1).

### 3.3. Retinoic Acid (Vitamin A)

The current definition of retinoid includes all the natural and synthetic compounds with an activity similar to that of Vitamin A. Vitamin A exists in the human body as various interconvertible compounds, notably retinal (essential for vision) and retinol, which is the most potent analogue and the main form of storage and transportation [27, 67]. Retinoic acid is obtained from carotene and animal products such as meat, milk, and eggs, which, while in the intestine, are converted, respectively, into retinal and retinol [27, 67]. The absorption of retinoids increases by up to 50% when ingested with food. Retinoids are transported in the blood by plasmatic proteins. Hepatic metabolization is achieved via the action of cytochrome P-450 [27, 67]. Hypervitaminosis occurs when consumption exceeds the liver's capacity to store retinoids [30].

Topic retinoids were initially tested against diseases related to keratinization. 13-cRA was used for the first time against acne, in 1969. The so-called “retinoic dermatitis” is the main side effect of tretinoin, this leads to cutaneous irritation characterized by erythema, scaling, ardency, and/or pruritus. “Retinoic dermatitis” occurs frequently, and patients ought to be previously instructed with regard to its occurrence. Furthermore, patients should also be warned to avoid the sunlight and to wear sunscreen [68].

The use of systemic retinoids is not indicated in cases of (1) pregnancy or probability of pregnancy; (2) noncompliance with the use of contraceptives; (3) breast feeding; (4) hypersensitivity to parabeno (in isotretinoin capsules). It is, relatively, not indicated in cases of (1) leukopenia; (2) hypothyroidism (patients using bexarotene); (3) high levels of cholesterol and triglyceride; (4) hepatic malfunction; (5) renal malfunction [67, 69]. Absorption of systemic retinoids is boosted by up to 60% when they are taken together with the meals.

Supplementation with retinoids for OL treatment began in the 1960s. However, this treatment was not widely accepted due to its side effects—hypervitaminosis, toxicity, teratogenic effects, and alterations in various organic systems [70]. At the cellular-level, retinoids interact with surface receptors and penetrate the cell. They are subsequently metabolized and transported to the nucleus by various proteins. Retinoids affect diverse processes, such as keratin production, the expression of growth factors and kinases, oncogenesis, apoptosis, production of the collagen matrix, immunologic and inflammatory response, cellular differentiation, embrionary morphogenesis and carcinogenesis [26, 30].

13-cRA is the retinoid recommended for OL treatment. The use of 13-cRA has been shown to be effective in resolving OL [33, 34]. However, the high recurrence rates after short periods of discontinuance, together with its side effects, are limiting factors [33, 34, 70]. Various studies have evaluated the therapeutic effectiveness of vitamin A derivatives in the treatment of OL, although not all studies have shown concordant results (Table 1). In one study, of the 45 patients registered, 7 (15.5%) had OL. Patients received a fixed dose of 13-cRA (10 mg/day) plus an escalating dose (beginning at 800 IU/day, until 2000 IU/day) for 4 months. Seventy-one percent of OL patients had complete clinical responses [71]. A study conducted with retinoic supplementation (300.000 IU retinol acetate) for OL treatment demonstrated complete resolution in 52% of patients. Side effects observed included six patients with headaches, five patients reported muscle pain, and two patients reported dry mouth [31].

Kaugars et al. [34] implemented retinoic supplementation in various dosages for OL treatment. Fifty percent of patients had complete or partial clinical resolution of OL, but with side effects such as dizziness and headache. Moreover, for most of the patients with clinical resolution of the lesion, OL recurred upon the discontinuance of medication. Some patients ceased treatment due to its side-effects. On the other hand, during the assessment of 13-cRA topical use (0.1% isotretinoin gel) for 4 months, in 9 patients with OL, 20% showed complete clinical response to treatment and no patient reported adverse effects [72].

In another study, 13-cRA was used in 16 patients with OL for six months. Three patients were entered at a dosage of 3 mg/day, eight at 5 mg/day, and five at 10 mg/day. Eleven patients completed the study: 3 had complete clinical responses (2 at 10 mg/day and 1 at 5 mg/day). Recurrence was observed in two of these three patients [73]. 

During another study, patients with OL were distributed into two groups: one receiving 200.000 IU vitamin A per week (*n* = 21) and the other receiving placebo capsules (*n* = 33) for six months. Complete remission was observed in 57% of patients that received vitamin A. The administered doses of vitamin A did not produce any detectable adverse effects during the trial period. In the placebo group, 7 patients (21%) formed new OL; whereas no new OL developed in the vitamin A group over the 6 months [74]. In an additional study from these same authors, patients with OL were divided into three groups receiving: group 1, beta-carotene (180 mg/week); group 2, beta-carotene (180 mg/week) plus vitamin A (100.000 IU/week), and group 3 placebo, for 6 months. Remission of OL in group 1 (14.8%) and group 2 (27.5%) differed significantly from that seen in group 3 (3%). During the trial period, all patients continued to chew tobacco-containing betel quids [75].

In a study by Toma et al. [76], sixteen patients with OL were treated with oral 13-cis-retinoic acid. The initial dose, given for 3 months, was 0.2 mg/kg/day, increasing by a further 0.2 mg/kg/day in successive 3 month cycles. The maximum dosage reached 1.0 mg/kg/day. Fourteen of the patients completed the trial and there was one complete response obtained at 0.4 mg/kg/day. After the retinoic acid treatment was stopped, patients were followed-up for 12 months; 2 patients showed regression of the responses obtained after 6 and 9 months [76]. 

One study, in two phases, was made in 70 patients with OL. In the first phase, the 67 patients were treated at a high dose of isotretinoin (1.5 mg/kg/day) for three months. Fifty-five percent of patients demonstrated complete or partial clinical responses. In the second phase of the study, 59 patients, with responses or stable lesions, were randomly assigned to maintenance therapy with either beta-carotene (30 mg/day; *n* = 33) or a low dose of isotretinoin (0.5 mg/kg/day; *n* = 26) for nine months. Twenty-two patients (92%) with isotretinoin and 13 patients (45%) with beta-carotene demonstrated a positive response [77].

Studies focusing on topical vitamin A and their derivates in the management of patients with OL have been reviewed by Gorsky and Epstein [23]. The use of topical tretinoin at 0.05% was evaluated in 26 patients with OL. Patients were followed for a mean of 23 months. Ten patients who had partial or no clinical response were submited at biopsies pre- and posttreatment, and the mean grade of histological features did not change. Twenty-seven percent of the patients had a complete clinical remission. Recurrence of OL was observed in approximately 40% of these patients after cessation of the applications. The use of topical vitamin A acid showed a limited effect in controlling OL [78]. 

In an open trial, the clinical efficacy of topical calcipotriol (vitamin D3 analogue) was compared with tretinoin in the therapy of hyperkeratotic oral lesions (leukoplakia). Forty patients had histologically proven OL, 20 were treated with calcipotriol (50 mg/g), and the other 20 with tretinoin cream (0.05%). The treatment was given for 5 weeks and follow-up was at 4 months, with clinical assessments at 2, 4, and 5 weeks. Results showed complete resolution of OL in 16 patients in both calcipotriol and tretinoin groups. No documented topical or systemic adverse reactions and results were maintained at 4 months [79].

In a 10-year study that followed OL patients, Scardina et al. [80] assessed the effectiveness of topical use of isotretinoin at 0.18%, as compared with 0.05%. Concentrations of 0.18% and 0.05% were given to two different groups and administered twice a day during 3 months. The clinical resolution was 85% in the 0.18% group, without any adverse topic or systemic reaction. In addition, epithelial dysplasia disappeared and there was a significant reduction in the size of the lesion.

In the systemic use with dosage of 300.000 IU of retinoic acid (Vitamin A), a clinical resolution of the 50% has been demonstrated. In topical use with dosage range from 0.05% to 1% a clinical resolution from 10% to 27% has been obtained (Table 1).

### 3.4. Fenretinide

Fenretinide (4-HPR) or N-(4-hydroxyphenyl) retinamide is a vitamin A analogue that was synthesized in the United States during the late 1960s. This retinoid shows a preferential accumulation in breast instead of liver [81], is effective in the inhibition of chemically induced mammary carcinoma in rats [82], and has proven to be less toxic than many other vitamin A analogues [82, 83]. A characteristic feature of 4-HPR is its ability to inhibit cell growth through the induction of apoptosis with mechanisms that may be both receptor-dependent and receptor-independent. Chemopreventive efficacy of fenretinide has been investigated in clinical trials targeted at different organs [84–86]. Eight patients with diffuse (nonoperable) oral lichen or OL were treated with 4-HPR applied topically twice daily. After one month of therapy, two patients had complete remission and the other six had a greater than 75% response. 4-HPR was well tolerated, and no local or distant side effects were observed [87].

A phase II trial of 4-HPR (200 mg/day) was carried out for 3 months in OL patients who had not responded (“de novo” resistance) or who had responded and then relapsed (acquired resistance) to the previous treatment with natural retinoids. Of 35 patients with retinoid-resistant OL, no patient had complete responses and 12 (34.3%) had partial responses to 4-HPR. Nine patients had clinical responses within 9 months of stopping 4-HPR. Toxicity was minimal and compliance was excellent [88].

Systemic use of 4-HPR with 200 mg/day for 3 months in 35 patients demonstrated partial clinical resolution of OL of 12 patients (Table 1).

## 4. Bleomycin

Bleomycin, a cytotoxic antibiotic, was first used for the treatment of neoplasms of the penis and scrotum, but has also been employed for squamous cell carcinoma of the head and neck region, oesophagus, and skin [89]. The most commonly adverse effects are mucocutaneous reactions, which include stomatitis, alopecia, pruritic erythema, and vesiculation of the skin [27]. Eight patients with OL were treated by the daily application of a 0.5% (w/v) solution of bleomycin sulphate in dimethyl sulphoxide (DMSO). After 12 to 15 applications, the white patch peeled off and the resultant raw suface was epithelialized over the following 14 days. Repeated biopsies showed a significant reduction of dysplasia and keratinisation [27]. The use of topical 1% bleomycin in DMSO was evaluated for the treatment of dysplastic OL. Bleomycin was applied once daily for 14 consecutive days to lesions of the oral mucosa in 19 patients. It was well tolerated with minor mucosal reactions. Immediate posttreatment biopsies showed that 75% of patients had resolution of dysplasia. Ninety-four percent of the patients attained at least partial clinical resolution. After a mean follow-up period of 3.4 years, 31.6% of patients had no clinically visible lesions. In 2 patients (11%), malignant transformation occurred [28].

Topical bleomycin in treatment of OL was used in dosages of 0.5%/day for 12 to 15 days or 1%/day for 14 days (Table 1).

## 5. Photodynamic Therapy

Photodynamic therapy (PDT) is a noninvasive method for the treatment of premalignant lesions and head and neck cancers [90, 91]. The principle of PDT is a nonthermal photochemical reaction, which requires the simultaneous presence of a photosensitising drug (photosensitiser), oxygen, and visible light. After a period to allow the photosensitiser to collect in the target tissue, the photosensitiser is activated by exposure to low-power visible light of a drug-specific wavelength. Mainly, the light source consists of a portable diode laser and the light is transmitted via laser fibres to or into the tumour. Illumination of the tumour by light at the activating wavelength results in the destruction of cells by a nonfree radical oxidative process. These reactive oxygen species may damage crucial cell components, such as structural proteins, enzymes, DNA, and phospholipids. PDT is a cold photochemical reaction, and the photosensitising agents are of inherently low systemic toxicity. PDT damage heals mainly by regeneration rather than scarring. Due to the organ preserving principle of PDT, important structures are maintained with good functional and cosmetic outcome [91, 92].

Several photosensitisers have been developed during the past. Haematoporphyrin and haematoporphyrin derivatives were the first photosensitisers. Four photosensitisers have been approved so far: (1) photofrin has been approved in many countries for the treatment of oesophagus cancer and lung cancer; (2) 5-Aminolaevulinic acid (ALA) was also approved in several countries for the treatment of skin cancer; (3) verteporfin for the treatment of macular degeneration (4) foscan is the only photosensitiser that has been approved for the treatment of advanced squamous cell carcinoma of the head and neck in Europe in the year 2001 [93].

The ALA is a naturally occurring compound in the haem biosynthetic pathway, which is metabolised to a photosensitive product, protoporphyrin IX (PpIX). The major advantage of ALA when compared to synthetic photosensitisers is the rapid metabolism, which significantly reduces the period of cutaneous photosensitivity [93]. For most indications in head and neck surgery, the photosensitiser is administered systemically by intravenous injection. Only for very superficial skin lesions or premalignant lesions of the oral mucosa, the ALA can be applied topically. For all other indications intravenous application is mandatory [93].

Zakrzewska et al. [94] reported three forms of treatment of 10 cases of proliferative verrucous leukoplakia; surgery, carbon dioxide laser; PDT. PDT was administered to 5 patients, in which there was no recurrence in 3, although a white halo of hyperqueratosis was observed around the area subjected to treatment. Results showed a recurrence rate, after treatment, of 100% for surgical excision and 85.7% for laser vaporization. PDT offered the best prognosis compared to the other forms of treatment. Kübler et al. [95] treated 20 patients with OL using PDT topic 20% ALA, followed by light jet at 630 nm, 100 W/cm2 and 100 J/cm2. After 3 months, 5 patients completely responded to the treatment (there were no clinical signs of OL), 4 partially responded (the lesion was reduced or looked better), 3 did not respond (no clinical change), and 1 had a partial response being submitted to treatment again, resulting in the disappearance of the lesion. No recurrence was observed at 9 months after this treatment.

Siéron et al. [90] treated 5 patients with OL using topical 10% ALA on the lesion, followed by argon laser (635 nm, 100–250 J/cm2). Four of the 5 patients completely responded. In one case, there was recurrence after 6 months, however, after 2 additional sessions, the lesions completely disappeared. This same author [96] observed the therapeutic response with PDT for OL in twelve patients topically treated with ALA at 10%, activated by a laser at 635 nm and 100 J/cm2 per session, for 6 to 8 sessions. There was a complete response (total wash out of the leukoplakia in the visual inspection confirmed by specimen biopsy) in 10 cases (83%). One recurrence was reported after 6 months of follow-up.

Chen et al. [97] treated 24 patients with OL using 20% ALA-PDT, once a week; another 24 patients used 20% ALA-PDT twice a week. In the latter group, 8 completely responded to the treatment, 16 partially responded, and 9 did not. All patients from the twice-a-week group responded significantly better than those treated only once a week.

From the studies using PDT-ALA in topical concentrations from 10 to 20%, it may be observed a clinical resolution of OL of the 25% to 80% (Table 1).

## 6. Conclusion

Several clinical trials have investigated the treatment of OL with use of supplements. Although the administration of retinoic acid and beta-carotene has some efficacy to resolve OL, the studies were based on small samples and short periods of follow-up. Given the side effects and counter-indications of antioxidizing agents, with the exception of lycopene, the use of agents requires careful control. The small number of patients, the lack of controls, the lack of widely accepted criteria for classifying OL, the variability in nonsurgical treatment protocols, and differences in histopathologic evaluation difficult the interpretation of data of the few randomized clinical trials in nonsurgical treatment of OL. At this time, randomized controlled trials for nonsurgical treatment of OL demonstrate no evidence of effective treatment in preventing malignant transformation and recurrence. It reinforces that after clinical resolution, OL should be regularly followed.

## Figures and Tables

**Table 1:** Nonsurgical treatments options for oral leukoplakia.

Author	Therapy	Dose	Number of patients	Clinical resolution (%)	Follow-up (months)	Recurrence	Malignant transformation (%)
Benner et al. [66]	Systemic Alfa tocoferol	400 IU	43	20%	24	NR	NR
Garewal et al. [38]	Systemic Beta-carotene	30 mg/day	24	8%	6	NR	8%
Toma et al. [37]	Systemic Beta-carotene	90 mg/day	23	26%	7	5%	NR
Sankaranarayanan et al. [31]	Systemic Beta-carotene	360 mg	46	54%	12	5%	NR
Liede et al. [32]	Systemic Beta-carotene	20 mg/day	24	NR	60–84	NR	NR
Garewal et al. [39]	Systemic Beta-carotene	60 mg/day	50	4%	18	17%	8%
Sankaranarayanan et al. [31]	Systemic Isotretinoin	300.000 IU	42	52%	12	67%	NR
Stich et al. [74]	Systemic Vitamin A	200.000–300.000 IU	21	57%	12	NR	NR
Nagao et al. [47]	Systemic Lycopene	NR	48	NR	NR	NR	NR
Singh et al. [48]	Systemic Lycopene	4–8 mg	58	25-55%	3	NR	NR
Hammersley et al. [24]	Topical Bleomycin	0,5%	8	NR	12	NR	NR
Epstein et al. [25]	Topical Bleomycin	1%	19	32%	40	NR	11%
Shah et al. [73]	Topical Vitamin A	1–5 mg	11	27%	11	18%	NR
Epstein and Gorsky [78]	Topical Tretinoin	0,05% gel	26	27%	42	40%	NR
Piattelli et al. [72]	Topical Isotretinoin	1%	10	10%	48	NR	NR
Lippman et al. [88]	Systemic 4 HPR	200 mg/day	35	0	9	NR	23%
Tradati et al. [87]	Topical 4 HPR	NR	8	25%	NR	NR	NR
Kübler et al. (1998)	Topical PDT	ALA 20%	20	25%	3	NR	NR
Siéron et al. [90]	Topical PDT	ALA 10%	5	80%	6	20%	NR
Sieroń et al. [96]	Topical PDT	ALA 10%	12	83%	6	8%	NR
Chen et al. [97]	Systemic PDT	ALA	24	33%	NR	NR	NR

IU: International Unit; NR: not reported; 4 HPR: Fenretinide; PDT: Photodynamic therapy; ALA: Aminolevulenic acid.
